# Specifically Targeting Capture and Photoinactivation of Viruses through Phosphatidylcholine-Ganglioside Vesicles with Photosensitizer

**DOI:** 10.1021/jacsau.4c00453

**Published:** 2024-08-01

**Authors:** Lenka Horníková, Petr Henke, Pavel Kubát, Jiří Mosinger

**Affiliations:** † Faculty of Science, BIOCEV, Charles University, Průmyslová 595, Vestec 252 50, Czech Republic; ‡ Faculty of Science, Charles University, Hlavova 2030, Prague 2 128 43, Czech Republic; # J. Heyrovský Institute of Physical Chemistry of the Czech Academy of Sciences, Dolejškova 3, Prague 8 182 23, Czech Republic

**Keywords:** singlet oxygen, photosensitizer, photodynamic, gangliosides, polyomavirus, photoinactivation

## Abstract

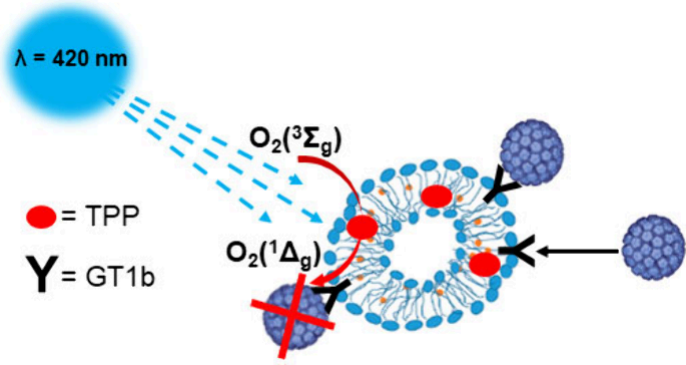

Herein, we performed a simple virus capture and photoinactivation procedure using visible light on phosphatidylcholine vesicles. l-α-Phosphatidylcholine vesicles were enriched by viral receptors, GT1b gangliosides, and the nonpolar photosensitizer 5,10,15,20-tetraphenylporphyrin. These vesicles absorb in the blue region of visible light with a high quantum yield of antiviral singlet oxygen, O_2_ (^1^Δ_g_). Through the successful incorporation of gangliosides into the structure of vesicles and the encapsulation of photosensitizers in their photoactive and monomeric state, the photogeneration of O_2_(^1^Δ_g_) was achieved with high efficiency on demand; this process was triggered by light, and specifically targeting/inactivating viruses were captured on ganglioside receptors due to the short lifetime (3.3 μs) and diffusion pathway (approximately 100 nm) of O_2_(^1^Δ_g_). Time-resolved and steady-state luminescence as well as absorption spectroscopy were used to monitor the photoactivity of the photosensitizer and the photogeneration of O_2_(^1^Δ_g_) on the surface of the vesicles. The capture of model mouse polyomavirus and its inactivation were achieved using immunofluorescence methods, and loss of infectivity toward mouse fibroblast 3T6 cells was detected.

## Introduction

Viruses are the most frequent causative agents of disease in humans and have a significant impact on morbidity and mortality worldwide. The viruses that most commonly circulate on all continents as endemic or epidemic agents are influenza virus, respiratory syncytial virus, parainfluenza virus, metapneumovirus, rhinovirus, coronaviruses, adenoviruses, and bocaviruses. Although much progress has been made in understanding the biology and fundamental aspects of the host–parasite relationship of these viruses, effective antiviral therapies are not available for most of these viruses.

In antiviral therapies, several strategies are used to fight viral infections. Since viruses, as obligate parasites, use the host cell machinery for many functions, antiviral drugs must be directed at the virus with care to prevent host cellular functions from being damaged. Therefore, antiviral drugs are designed to target viruses, especially virus-encoded enzymes, e.g., polymerases or proteases. As another option, drugs such as antibodies^1^ or aptamers^2^ have been developed. Although these compounds bind virions and prevent viruses from entering host cells, they cannot directly inactivate the virus.

In recent years, a potential alternative to inactivate viruses has emerged called singlet oxygen, O_2_(^1^Δ_g_), which is a short-lived, highly oxidative, and cytotoxic species; O_2_(^1^Δ_g_), is generated in situ by energy transfer from the excited triplet state of photosensitizer (^3^PS) to the ground-state triplet oxygen O_2_(^3^Σ_g_^–^) coupled with spin inversion of oxygen to O_2_(^1^Δ_g_) (eq 1).^3^
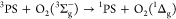
1Some nonpolar PSs can be incorporated in transparent carriers with sufficient oxygen diffusion to protect excited states from aggregation and quenching in aqueous media. When exposed to light, photoproduced O_2_(^1^Δ_g_) diffuses from the PS to the surface and shows the potential to kill microorganisms on the carrier surface or nearby areas.^4,5^ The radial diffusion distance traveled by O_2_(^1^Δ_g_), *d*, can be expressed as follows:
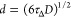
2where *D* is the oxygen diffusion coefficient in the carrier. During the O_2_(^1^Δ_g_) lifetime (τ_Δ_) the oxygen molecules can typically diffuse beyond tens to hundreds of nm, indicating that the contribution of O_2_(^1^Δ_g_) formed deeper from the surface to the antiviral properties is negligible.

The main drawbacks of these carriers with encapsulated photosensitizers are the short lifetime (τ_Δ_ ∼ 3.5 μs in aqueous media) and the diffusion length of O_2_(^1^Δ_g_); thus, efficient photooxidation of chemical and biological targets can occur only in areas near the surfaces.^6^ This drawback can be overcome by nanocarriers with large surface-to-volume ratios and/or with surfaces that efficiently bind pathogens such as specific pathogen receptors.

As a model virus, mouse polyomavirus (MPyV) was used in this study. MPyV is a small nonenveloped DNA virus that belongs to the *Polyomaviridae* family. Viruses from this family infect a wide range of hosts, including humans. Although the seroprevalence in the human population is high,^7^ asymptomatic infections with polyomaviruses in humans are common. However, in immunocompromised hosts, these viruses can cause severe diseases, such as polyomavirus-associated nephropathy (BK polyomavirus),^8^ progressive multifocal leukoencephalopathy (JC polyomavirus),^9^ or Merkel cell carcinoma (Merkel cell polyomavirus).^10^ Polyomaviruses are a considerable hurdle for the growing number of immunotherapies because polyomaviruses are a rapidly expanding family with high seroprevalence.^11,12^ Many polyomaviruses use glycans terminated in the sialic acid N-acetyl neuraminic acid^13^ as receptors for cell entry. Several gangliosides have been identified as attachment factors for polyomaviruses.^14^ Gangliosides GD1a, GT1b,^15^ and GT1a^16^ were identified as receptors for MPyV. Like MPyV, ganglioside GT1b is recognized by BK polyomavirus, and Merkel cell polyomavirus and BK virus can also bind GD1b and gangliosides GD2 and GD3.^17^

This work describes the preparation and properties of L-α-phosphatidylcholine vesicles with viral receptors encapsulating porphyrin photosensitizers. Through incorporating gangliosides into the structure of vesicles together with the encapsulation of 5,10,15,20-tetraphenylporphyrin (TPP), the photogeneration of O_2_(^1^Δ_g_) can be achieved with high efficiency and antiviral activity, specifically targeting viruses captured on ganglioside receptors.

## Results and Discussion

To demonstrate our concept, we searched for the most simplified system that achieves close contact between gangliosides and photosensitizers. l-α-Phosphatidylcholine (SPC) vesicles were selected as the TPP photosensitizer carriers. Through the self-incorporation of GT1b gangliosides into these vesicles, controls were constructed, both without gangliosides and without TPP. Although vesicle size can influence relative photooxidation efficiency, our emphasis was not on achieving monodispersity and small vesicle size but rather on maintaining critical parameters upon the encapsulation of TPP or the integration of gangliosides. The vesicle size and concentration were characterized by using DLS.

All the samples exhibited two main populations of vesicles, smaller with a diameter of approximately 41 ± 6 nm and larger with a diameter of 120 ± 40 nm. The particle concentrations per milliliter that were estimated from DLS were 10^12^ for the 40 nm vesicles and 10^11^ for the 120 nm vesicles.

**Figure 1:**
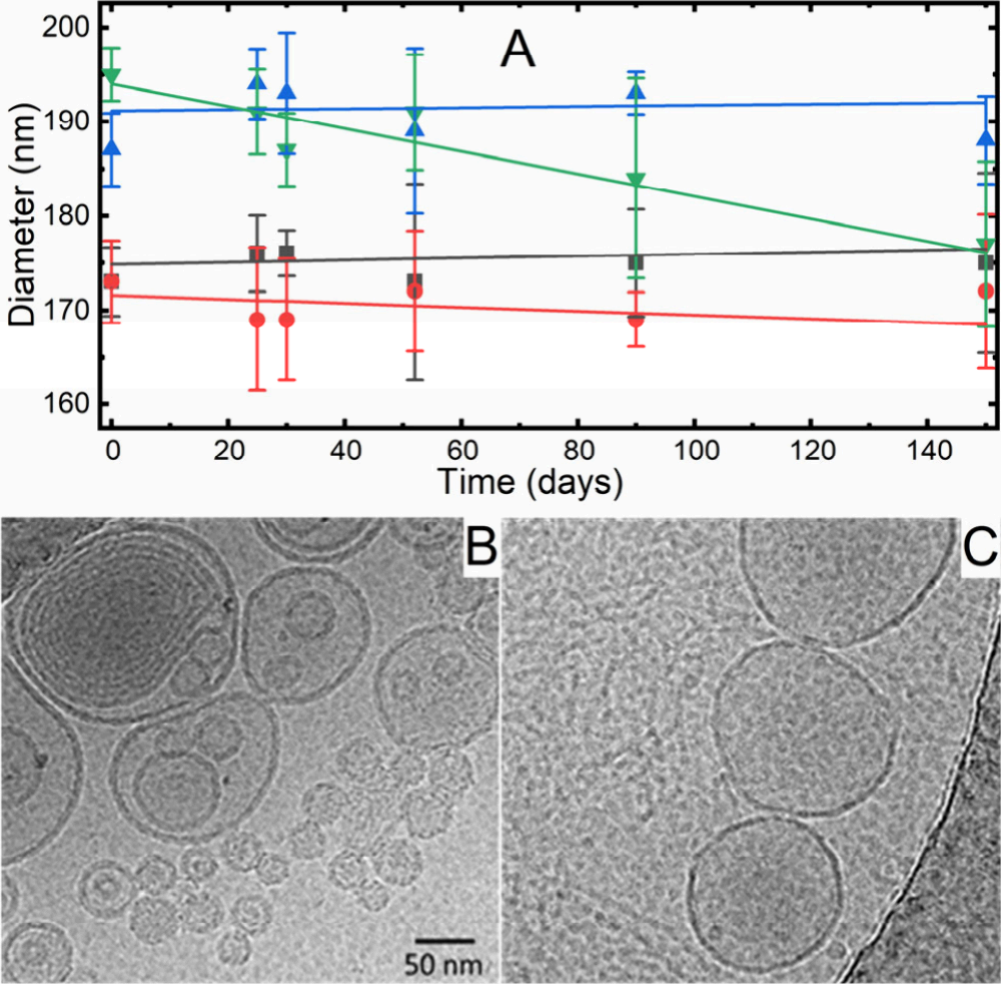
(A) Stability of the vesicle diameters over time during storage in a refrigerator for **1** (blue), **1@TPP** (red), **2** (green), and **2@TPP** (black). Each point represents the average diameter with the highest intensity from DLS measurements from at least three measurements. TEM (cryoEM) images of (B) **2@TPP** and (C) **1** illustrating the difference observed between samples with and without TPP.

Even after 150 days of storage in the refrigerator, the average size and estimated concentration of both vesicle populations remained practically unchanged (Figure 1A).

Despite consistent sizes and populations without and with GT1b (**1** and **2**), differences were observed between samples with (**1@TPP** and **2@TPP**) and without TPP (**1** and **2**) when analyzed using CryoEM; these results revealed that vesicles without TPP (**1**) were less populated and exclusively unilamellar, and vesicles with signs of rupture were observed only from the larger population (100 nm plus). In contrast, the sizes of the **1@TPP** and **2@TPP** vesicles were less than 50 and greater than 100 nm, respectively, and the vesicles were predominantly multilamellar, typically consisting of several layers with 2–3 vesicles (Figure 1B, C).

**Figure 2:**
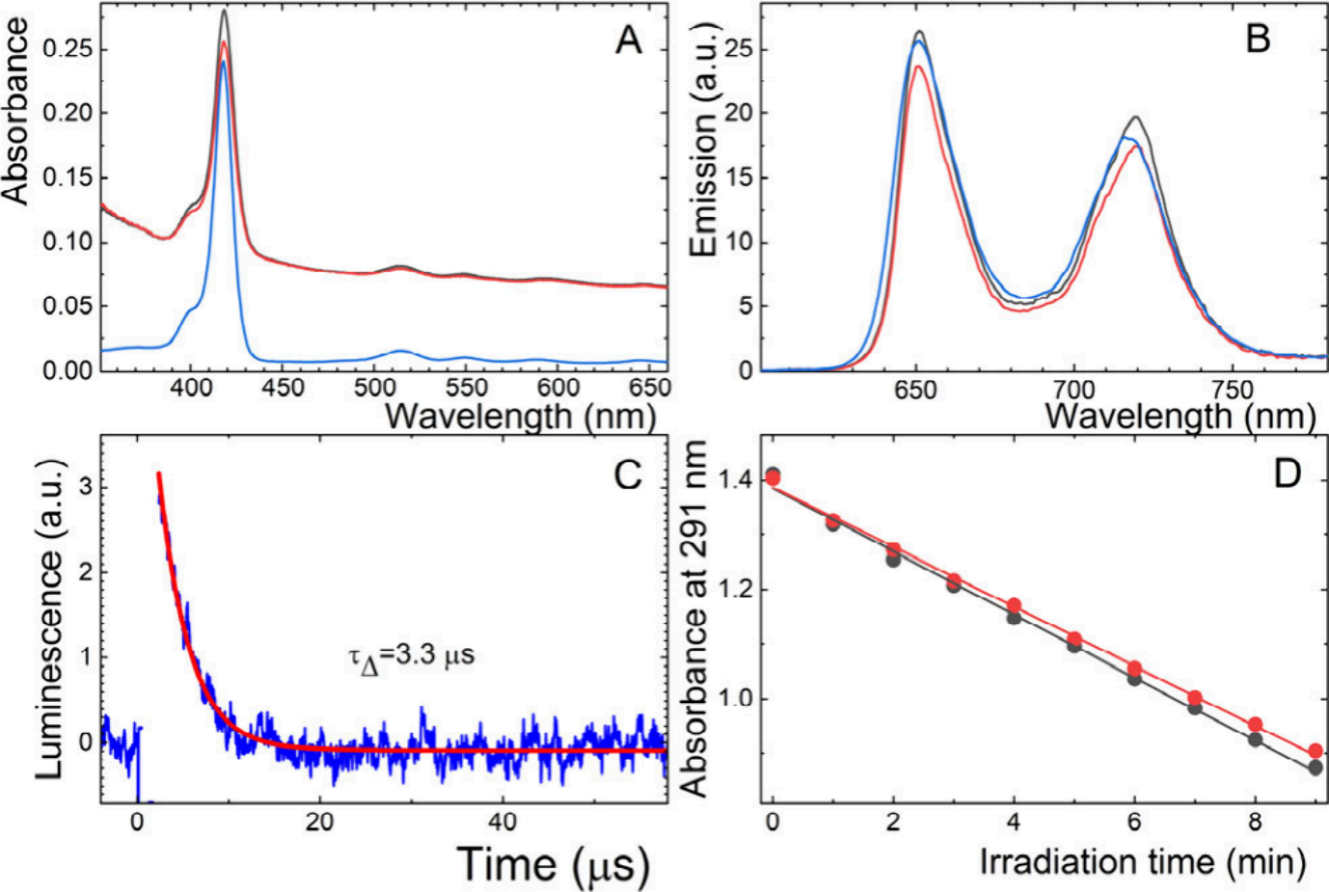
(A) UV/vis absorption and (B) fluorescence spectra of **1@TPP** (black), **2@TPP** (red), and 0.5 μM TPP in CHCl_3_ (blue). (C) Luminescence of O_2_(^1^Δ_g_) at 1270 nm for the oxygen-saturated **2@TPP** dispersion after excitation by an excimer laser. The red line is a single exponential fit to the experimental data. (D) Photooxidation efficacy of uric acid sodium salt by O_2_(^1^Δ_g_) represented by a linear fit through changes in the absorbance of uric acid at 291 nm after irradiation by a 500 W Xe lamp of **1@TPP** (black) and **2@TPP** (red).

UV/vis absorption and emission spectra (Figure 2A, B) show that TPP in both **1@TPP** and **2@TPP** is not aggregated. The aggregation of TPP, which is typical in polar environments, is accompanied by broadening and hypochromicity of the Soret band, quenching of excited states, and O_2_(^1^Δ_g_) photogeneration.^18^ The absence of these effects in the spectra strongly suggested that nonpolar TPP molecules are dispersed within the vesicles or in the nonpolar region of the phospholipid layers.

Next, O_2_(^1^Δ_g_) luminescence was directly measured to determine whether adding gangliosides to phospholipid vesicles influences O_2_(^1^Δ_g_) photogeneration and lifetime. The kinetic profiles of the luminescence were fitted to a single-exponential decay function for the calculation of the lifetime of the O_2_(^1^Δ_g_) lifetime (τ_Δ_). The calculated values of τ_Δ_ for both **1@TPP** and **2@TPP** (3.3 μs, Figure 2C) are close to the reported value of 3.5 μs in water^19^ and far from the 14 μs observed for phosphatidylcholine.^20^ This suggests that TPP is close to the surface and in situ created O_2_(^1^Δ_g_) readily escaped to the aqueous environment.

The kinetics of the TPP triplet states for **2@TPP** were measured by transient absorption spectroscopy from the changes in absorbance at the triplet–triplet absorption band of TPP at 460 nm. The lifetime of the triplet states (Figure S1 and Table S1) and the fraction of the TPP triplet states quenched by oxygen in air-saturated dispersions (*F*_T_^air^ = 0.94) show that the TPP triplets were efficiently quenched by oxygen, leading to the formation of O_2_(^1^Δ_g_) and excellent oxygen access to the TPP triplets.

To determine whether O_2_(^1^Δ_g_), a species with a short lifetime and diffusion pathway, can escape from vesicles to reach a target in aqueous media, a chemical substrate test was performed. A uric acid sodium salt (UA)-specific probe for O_2_(^1^Δ_g_) was selected for the test. UA undergoes selective oxidation by O_2_(^1^Δ_g_), and the kinetics of its bleaching were observed during irradiation by UV/vis spectroscopy. The relative photooxidation ability of each sample was determined by analyzing the slope of the kinetics of photobleaching, as illustrated in Figure 2D. Only a small difference in the slopes of the kinetics for samples **1@TPP** and **2@TPP** (∼5%) was observed.

This variation falls within the range of experimental error and is consistent with the ∼7% lower absorbance of **2@TPP** than that of **1@TPP** (Figure 2A). This close alignment between the two measurements reinforces the reliability of our experimental findings. The small difference (∼5%) in O_2_(^1^Δ_g_) production between samples with and without gangliosides is negligible, particularly considering the context of our study, which involved biological evaluations.

**Figure 3:**
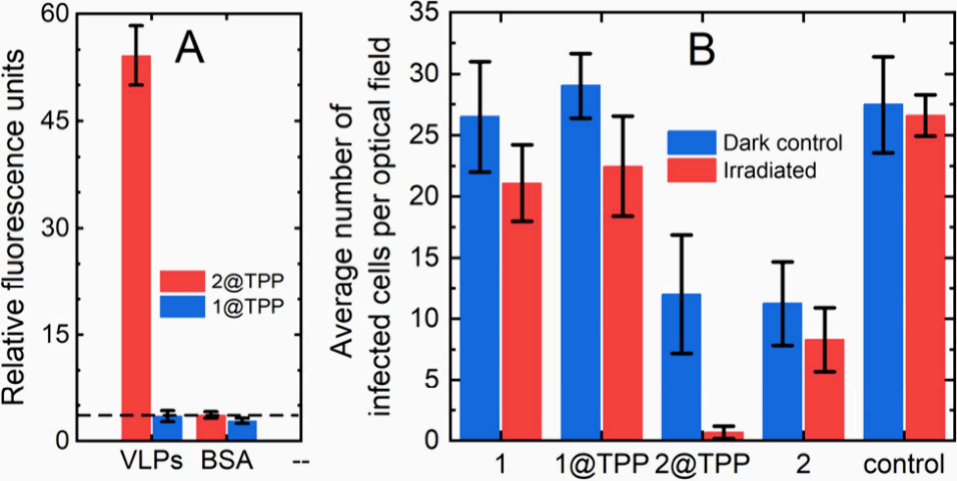
(A) Demonstration of the specific binding of VLPs to **2@TPP** compared to that of the controls (VLPs to **1@TPP**, both to BSA) using a modified ELISA with fluorescence detection. The dashed line represents the background fluorescence value of PBS. (B) Visible light-induced antiviral activity of **2@TPP** in contrast to that of the controls (**1**, **1@TPP**, **2**, and PBS). Samples **1**, **2**, **1@TPP**, **2@TPP**, or PBS were mixed with virus and incubated for 1 h at constant agitation. Then, the samples were diluted two times, irradiated for 5 min, or kept in the dark, after which the cells were infected. Cells were fixed 24 h post infection, and infected cells were stained with specific antibodies. The values in the graphs refer to the numbers of infected cells per optical field and represent the mean values from four independent experiments ± SDs. For experimental details see the Supporting Information.

To test whether vesicles with anchored gangliosides bind mouse polyomavirus particles, a modified ELISA was performed. The wells were coated with VLPs instead of virus particles because they are more easily produced and exhibit the same behavior in receptor binding and trafficking within the cell.^21,22^ Then, the wells were incubated with **1@TPP** or **2@TPP,** and the ability of VLPs to interact with vesicles was assessed indirectly by measuring the TPP fluorescence in the wells. VLPs captured sample **2@TPP** but not sample **1@TPP**. The fluorescence measured was at the same level as the background fluorescence. The interaction was further verified by testing the binding of sample **2@TPP** and sample **1@TPP** to an irrelevant protein, bovine serum albumin (BSA). None of the vesicles used interacted with BSA, and the fluorescence values were comparable to those of the background signal (Figure 3A).

To evaluate whether the antiviral activity of TPP may be targeted to a specific virus, vesicles (**1**, **1@TPP**, **2** and **2@TPP**) or PBS (control) was mixed with MPyV, and the virus was allowed to bind to the vesicles for 1 h. However, because the antiviral effect of O_2_(^1^Δ_g_) is effective only at limited distances, the mixtures were diluted to prevent the virus from being randomly activated by TPP from the unbound vesicles. Mixtures were kept in the dark or irradiated with light for 5 min and were then applied to 3T6 fibroblasts. Virus infectivity was analyzed by the use of specific antibodies directed against virus proteins.

Figure 3B shows the complex effect of the samples on virus infectivity combined with the photoinduced antiviral effect and the binding of viruses to vesicles. Photoinduced antiviral effects accompanied by a loss of infectivity can be observed in samples containing TPP, **1@TPP**, and especially **2@TPP**, which bind viruses on surfaces. However, light itself had a slight antiviral effect,^23,24^ even without a photosensitizer. The significant effect on the loss of viral infectivity can also be attributed to the profound binding of viruses to vesicles containing gangliosides (**2** and **2@TPP**). These vesicles may mimic soluble ganglioside, which has been shown to block viral infection by preventing viral binding to cells.^15^ Concurrently, complexes involving multiple viruses bound on a single vesicle may form. This complex may be less likely to enter and infect cells or result in a scenario in which one infection occurs instead of several infections. This interpretation is supported by the pronounced dark antiviral effect observed in samples **2** and **2@TPP**.

In summary, these data show that antiviral activity is increased by the specific interaction between viral particles and viral receptors on the surface of vesicles with encapsulated TPP. The specific and tight interaction allows virus particles to remain in close proximity and concentrate at the vesicle surface. Together, these results ensure the efficient antiviral activity of TPP. Moreover, vesicles containing virus receptors or other molecules may be used for targeting TPP activity.

## Conclusion

Herein, we demonstrate the simple preparation of photoactive L-α-phosphatidylcholine vesicles with viral ganglioside receptors that can encapsulate the monomeric form of the tetraphenylporphyrin photosensitizer with a high quantum yield of antiviral O_2_(^1^Δ_g_). This construct allows the photogeneration of O_2_(^1^Δ_g_) with high effectiveness and antiviral activity that specifically targets viruses captured on ganglioside receptors. This specificity is supported by the light-targeting absorption band of the photosensitizer used and the limited diffusion pathway of the photogenerated O_2_(^1^Δ_g_) in close proximity to the virus captured on the receptors. Photoactive antiviral vesicles are promising for numerous applications, especially in the biomedical field, in which viral capture with local antiviral effects can be applied using visible light.

## Methods

### Cells and Virus

Mouse fibroblast 3T6 cells (ATCC; CCL-96) were grown at 37 °C in a 5% CO_2_-air humidified incubator using Dulbecco’s Modified Eagle Medium (DMEM; Merck) supplemented with 10% bovine serum (Merck). Sf9 (*Spodoptera frugiperda)* insect cells (ATCC; CRL-1711) were cultivated as adherent cultures at 27 °C in an Insect-XPRESS Protein-free Insect Cell Medium (Lonza). Using standard protocol,^25^ mouse polyomavirus strain BG (GenBank: J02289.1) was isolated and purified from infected 3T6 cells. Recombinant baculovirus AcDB3/VP1/EGFP-t-VP3 was propagated using the standard protocol.^22^

### Antibodies

The primary antibody used in the study was a rat monoclonal antibody to large T protein,^22^ and the secondary antibody was a goat antirat antibody conjugated with Alexa Fluor-488 (Thermo Fisher Scientific).

### Virus-like Particle Isolation

The cells were infected with multiplicity of infection 10 plaque forming units per cell and harvested at 72 h post infection (hpi). VP1/EGFP-tVP3 virus like particles (VLPs) were isolated using the standard protocol^26^ and protein concentration of isolate was determined by Rapid Gold BCA Protein Assay Kit (Thermo Fisher Scientific) according to manufacturer̀s protocol.

### Preparation of Multilamellar Vesicles

l-α-Phosphatidylcholine (SPC) in the amount of 15 mg was dissolved in 1 mL of chloroform with or without 0.15 mg of TPP that was subsequently evaporated in nitrogen blow at a temperature of 4 °C. Large multilamellar vesicles were prepared by intensive shaking of this sample with 5 mL of PBS. The resulting suspension had 3 mg/mL. To prepare the final vesicles, the suspension was sonicated and tempered to 50 °C. After that, the suspension was filtrated thrice through a 200 nm filter while still tempered to 50 °C. According to UV–vis measurements, the resulting vesicles (**1**) with TPP (**1@TPP**) lost 2/3 of TPP during the process. Mixed vesicles with ganglioside with and without TPP (**2@TPP** respective **2**) were prepared by adding 1 mg of GT1b ganglioside to 5 mL of **1** or **1@TPP** and with subsequent 1 h sonication and overnight incubation tempered to 50 °C.

### Specific Binding of Vesicles to Mouse Polyomavirus Particles

Wells of microtitration plate for enzyme linked immunosorbent assay (Thermo Fisher Scientific) were coated with 0.5 μg of VLPs (∼2 × 10^10^ particles) or 0.5 μg of bovine serum albumin in PBS at 4 °C overnight. Between each step, the plate was washed 3 times with PBS. Wells were blocked with 200 μL/well of 2% nonfat milk in PBS for 2 h. Vesicles with gangliosides containing TPP (**2@TPP**) or not (**2**) were eight times diluted, and 100 μL/well of diluted sample was incubated on a plate protected from light with constant agitation at room temperature overnight. Fluorescence was measured in PBS at 419 nm/651 nm (excitation/emission) using Infinite M Plex (Tecan). The samples were run in duplicates and PBS was used for background fluorescence assessment.

### Test of Antiviral Activity of Vesicles

In the well of 24 well dish (SPL) was mixed virus inoculum (5 × 10^4^ ffu; ∼2 × 10^10^ virus particles) with 10 μL (∼10^7^ particles) of vesicles in total volume 200 μL of DMEM media. Samples were incubated 1h at room temperature with constant agitation and protected from light. Then, samples were diluted with 200 μL DMEM media, placed on the ice, and irradiated 5 min with LED lamp or kept in the dark. The LED lamp contained 18 1W LED diodes and was placed in 30 cm distance with measured output of 3.6 J s^–1^ cm^–2^ for maximum λ in 423 nm (ILT960 spectroradiometer SpectriLight from International Light Technologies, USA). The medium containing viruses and vesicles was then used for infection. Mouse fibroblast cells (1 × 10^5^) growing on coverslips were washed with DMEM and 200 μL of media containing virus was adsorbed to the cell surface for 1 h at 37 °C in a 5% CO_2_-air humidified incubator. Then, 800 μL of prewarmed DMEM media supplemented with 10% bovine serum was added and incubated for 24 h. Infected cells were washed in PBS, fixed in 3.7% paraformaldehyde in PBS for 15 min, permeabilized by 0.5% Triton X-100 in PBS for 5 min, and washed in PBS three times. Then, the samples were blocked with 0.25% gelatin and 0.25% bovine serum albumin in PBS for 30 min. Immunostaining with primary and secondary antibodies was carried out for 1 h and 30 min, respectively. After both primary and secondary antibody incubation, the samples were extensively washed (3 × 10 min) with PBS. Finally, the samples were mounted on droplets of DAPI Gold solution (Thermo Fisher Scientific). The samples were observed by using an Olympus IX73 microscope. The numbers of infected cells were scored by immunofluorescence microscopy. At least 300 cells were counted for each experiment. The amount of the infected cells of the irradiated samples was compared with those of unirradiated samples.

Other experimental details (characterization of vesicles and photophysical properties) are available in the Supporting Information and in our previous papers.^5,23,24^

## Supporting Information Available

Experimental details and photophysical measurements (PDF)
