# Supplementary material for: Specifically Targeting Capture and Photoinactivation of Viruses through Phosphatidylcholine-Ganglioside Vesicles with Photosensitizer
Source: JACS Au. 2024 Aug 1;4(8):2826–31. doi: 10.1021/jacsau.4c00453 (PMC11350727; doi:10.1021/jacsau.4c00453)
Supplement: Supplementary file 1 — au4c00453_si_001.pdf [file au4c00453_si_001.pdf]

Supporting Information

Specifically Targeting Capture and Photoinactivation of Viruses through Phosphatidylcholine-Ganglioside Vesicles with Photosensitizer

Lenka Horníková,[†] Petr Henke,[‡] Pavel Kubát,[#] and Jiří Mosinger [‡*]

[†] *Faculty of Science, BIOCEV, Charles University, Průmyslová 595, Vestec, Czech Republic*

[‡] *Faculty of Science, Charles University, Hlavova 2030, 128 43 Prague 2, Czech Republic*

[#] *J. Heyrovský Institute of Physical Chemistry of the Czech Academy of Sciences, Dolejškova 3, 182 23 Prague 8, Czech Republic*

Experimental Details

Chemicals. 5,10,15,20-Tetraphenylporphyrin (TPP), uric acid sodium salt, GT1b ganglioside, L-α-Phosphatidylcholine (SPC), and chloroform were purchased from Sigma-Aldrich. Phosphate buffer saline (PBS) was purchased from Lonza.

Dynamic light scattering. The size of the vesicles and particle concentration were measured using a Zetasizer Ultra ZSU5700 (Malvern).

Cryogenic electron microscopy (CryoEM). Samples for CryoEM analysis were frozen using the Leica GP2 Plunge Freezer. The samples, dispersed in PBS, were applied to grids (Quantifoil R 1.2/1.3, 300 mesh, glow discharge treated using the Henniker Plasma HPT-100) at room temperature and 80% relative humidity, and then plunge-frozen in liquid ethane at -180°C. CryoEM analysis was conducted on a Jeol JEM2100Plus TEM microscope equipped with a LaB6 electron gun and a TVIPS XF416 camera. Data were acquired in a semi-automated manner using SerialEM software[1] with a pixel size of 0.231 nm, an exposure time of 3 s, and an electron dose of 45 e/A^2.

UV−Vis Absorption and Emission Spectroscopy. UV−vis absorption spectra were collected on Unicam 340 and Varian 4000 spectrometers equipped with an integration sphere. Steady-state fluorescence spectra were obtained on an FLS 980 spectrofluorometer (Edinburgh Instruments).

Photooxidation of Uric Acid sodium salt. During the test, 2.65 mL of PBS and 100 µL of 1@TPP or 2@TPP were mixed with 250 µL of 10^{-3} M uric acid sodium salt solution in 1 cm quarts’ cuvette. The resulting suspension was either kept in the dark or irradiated in a tempered (25°C) holder with a 500 W Xenon lamp equipped with a 400 nm cut-on filter (Newport). The changes in UV−vis absorbance at 291 nm were monitored at regular intervals.

Photophysical properties. Time-resolved, near-infrared luminescence of O_2 ($^1\Delta_g$) at 1270 nm after excitation by an excimer laser (wavelength of 308 nm, pulse length of 28 ns) was observed using a homemade detector unit (Ge diode Judson J16-8SP-R05M-HS with an amplifier) and averaged to increase the signal-to-noise ratio. A long-pass filter (λ>1000 nm) and band-pass interference filter (λ~1270 nm) were placed between the sample and the detector. The luminescence of O_2 ($^1\Delta_g$) was corrected to the background luminescence in an argon-saturated dispersion, where no O_2 ($^1\Delta_g$) was formed. The temporal profiles of the luminescence were fitted to a single-exponential decay function (for calculation of τ_Δ) with the exclusion of the initial portion of the plot, which was affected by light scattering, TPP fluorescence, and kinetics of the TPP triplet state deactivation.

The kinetics of the triplet states were measured by transient absorption spectroscopy from the changes of absorbance at the triplet-triplet absorption band of TPP (460 nm) after excitation by an excimer laser using an LKS 20 laser kinetic spectrometer (Applied Photophysics, UK) equipped with an R928 photomultiplier (Hamamatsu). Where appropriate, the samples were saturated by oxygen and argon. A single exponential function fitted the kinetic traces to calculate the lifetime of the triplet states (τ_T). The fraction of the TPP triplet states quenched by oxygen in air-saturated dispersions was calculated asF_T^{air} = 1 - τ_T/τ_{TAr}, where τ_T and τ_{TAr} are the lifetimes of the triplet states in air- and argon-saturated dispersions, respectively.

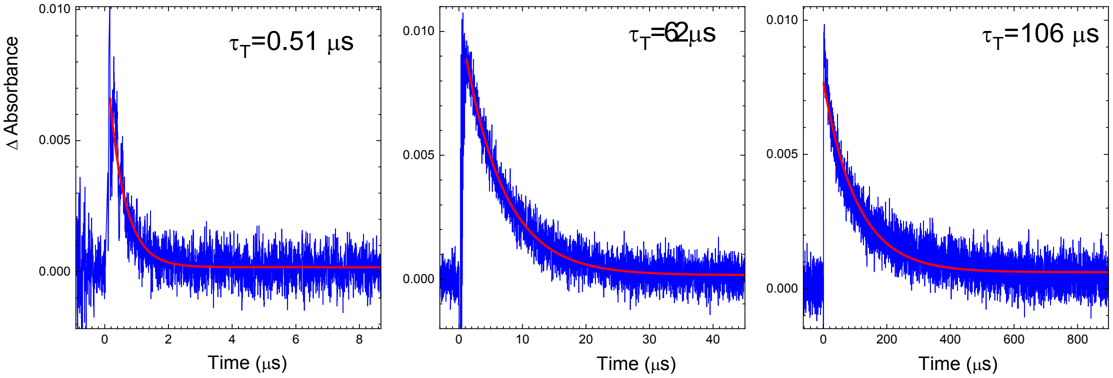

Figure S1. Kinetics of the TPP triplet states deactivation measured by transient absorption at 460 nm in oxygen- air- and argon-saturated **2@TPP**. Red lines are single exponential fits to the experimental data.

Table S1. The data from photophysics measurements

	τ_Δ	τ_T			F_T^{oxygen}	F_T^{air}
		oxygen	air	argon		
**2@TPP**	3.3	0.51	6.2	106	0.99	0.94
**1@TPP**	3.3	-	-	-	-	-

References

(1) Mastronarde, D. N., Automated electron microscope tomography using robust prediction of specimen movements. *J. Struct. Biol.* **2005,** *152*, 36-51.
